# Description of three species of ophioplinthacids, including a new species, from a deep seamount in the Northwest Pacific Ocean

**DOI:** 10.7717/peerj.11566

**Published:** 2021-07-02

**Authors:** Wanying Chen, Jieying Na, Dongsheng Zhang

**Affiliations:** 1 Key Laboratory of Marine Ecosystem Dynamics, Second Institute of Oceanography, Ministry of Natural Resources, Hangzhou, China; 2 Southern Marine Science and Engineering Guangdong Laboratory (Zhuhai), Zhuhai, China; 3 School of Oceanography, Shanghai Jiao Tong University, Shanghai, China

**Keywords:** *Ophioplinthaca*, New species, Taxonomy, Seamount, The Northwest Pacific

## Abstract

Five specimens of brittle star were collected from a deep-sea seamount in the Northwest Pacific, and identified into three species. One which is new to science, *Ophioplinthaca grandisquama* n. sp., can be easily distinguished from its congeners by the distinctly elongated and stout tentacle scales, stout and long disc spines, capitate with typically elongate to flaring head bearing numerous distinct thorns, radial shields roughly triangular and contiguous distally. One specimen was identified as *Ophioplinthaca semele* (Clark, 1949), which had been reported in Hawaii seamounts, is a new record of this species in the Northwest Pacific. The remaining specimen was an unknown species of *Ophioplinthaca*, with some different characteristics from other species of *Ophioplinthaca*. However, we, herein, prefer not to attach a name to this specimen until more morphological characteristics are available. The finding of this new species and two new records further enriches the distribution of *Ophioplinthaca* in the seamount of Northwest Pacific, providing useful information for marine protection in the cobalt-rich area.

## Introduction

*Ophioplinthaca* Verrill, 1899 is a genus in the family Ophiacanthidae Ljungman, 1867 which is distinguished from other Ophiacanthid genera by the deep interradial incisions into the disc which are lined distally by enlarged disc plates (O’Hara & Stöhr, 2006). *Ophioplinthaca* is a widely distributed genus, and according to WoRMS (Stöhr, O’Hara & Thuy, 2021), thirty-one valid species are known around the world. Among which, twenty-one species have been found occurring in the Indo-Pacific Ocean, six in the West Indian Ocean, and seven in the Atlantic Ocean (Cherbonnier & Sibuet, 1972; O’Hara & Stöhr, 2006; Clark, 1949; Clark, 1939; Clark, 1911; Lyman, 1878; Thomson, 1877; Koehler, 1904; Koehler, 1930; Koehler, 1922; John & Clark, 1954; Lyman, 1883; Guille, 1981; Koehler, 1897; Mortensen, 1933; Clark, 1900). Recently, it was suggested that *Ophioplinthaca* is one of the dominant groups of megafauna on seamounts (O’Hara, Rowden & Williams, 2008; Cho & Shank, 2010). The northwest Pacific region has the highest number of seamounts globally (Yesson et al., 2011), and many of the seamounts are covered with cobalt-rich ferromanganese crusts, which is a valuable mineral (Hein, Conrad & Dunham, 2009). However, few studies of Ophiuroid in this area has been reported (Litvinova, 1981; Zhang et al., 2018; Na et al., 2019).

In 2019, several *Ophioplinthaca* specimens were collected from RC seamount in the Northwest Pacific by a Remotely Operated Vehicle (ROV). Three specimens were determined to be a new species of genus *Ophioplinthaca* which we described herein. The other two specimens, identified as *Ophioplinthaca semele* and an unknown species, were described here as new records of ophioplinthacids in the Northwest Pacific Ocean. This study provides biodiversity information of seamounts in the cobalt-rich area, which may be useful for marine protection from future deep-sea mining.

## Materials & Methods

**Figure 1:**
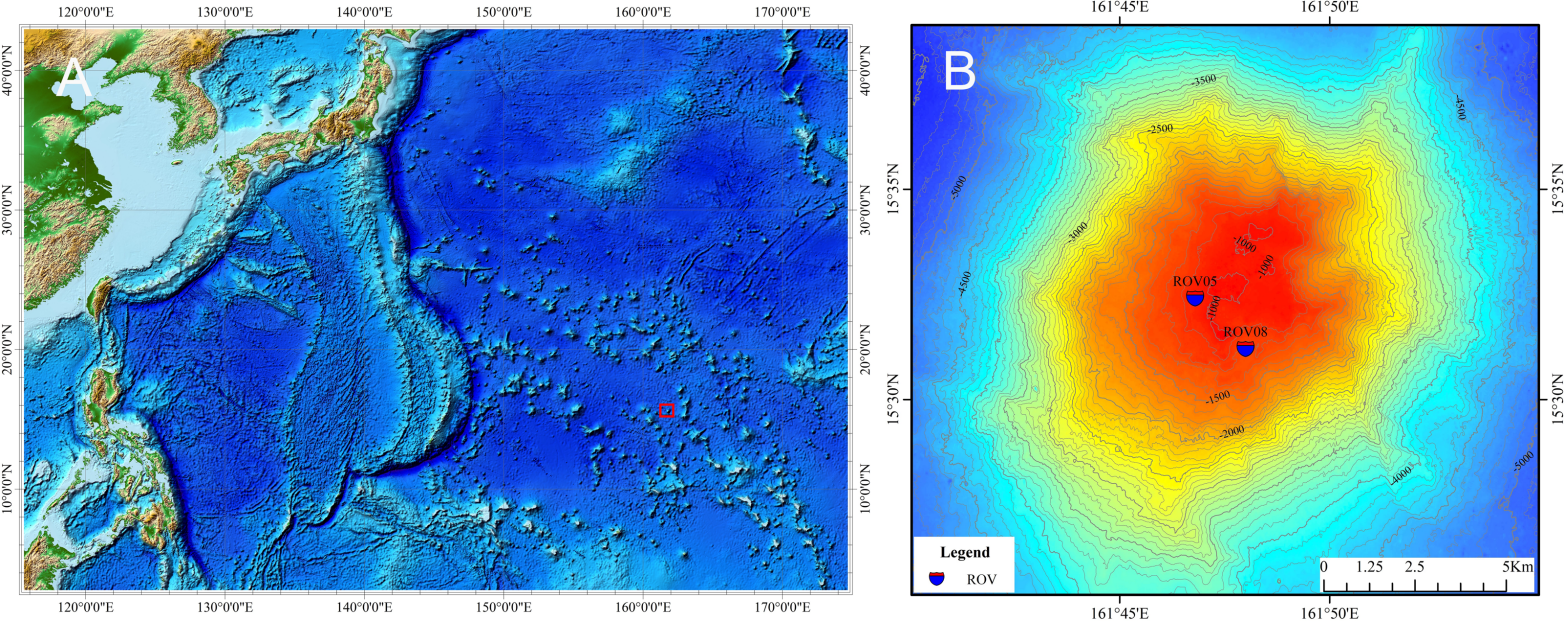
Map of the study seamount (indicated by the small red block) in the northwest Pacific (A) and sampling sites of specimens of ophioplinthacids (B). Credit attribution: Dr. Lin Shiquan.

Ophiuroid specimens from a seamount in the Northwest Pacific Ocean were collected during cruise DY56 using an ROV *HAILONG III*. Sampling sites are shown in Fig. 1. Specimens were fixed in 90% ethanol on board and deposited in the sample Repository of the Second Institute of Oceanography (RSIO), Ministry of Natural Resources, Hangzhou, China.

Specimens were examined and photographed using a stereoscopic microscope (Zeiss Axio Zoom.V16). Arm skeletal elements were obtained after submerging in commercial bleach (2.5% NaOCl), until all soft issue dissolved, washed in distilled water, air-dried and then mounted on stubs, imaged using a Hitachi TM1000 scanning electron microscope.

Genomic DNA was extracted from arm tissue using DNeasy^®^ Blood & Tissue Kit (QIAGEN) following the manufacturers’ protocols. The mitochondrial COI sequences were amplified with primers listed in Table 1. PCR reactions were performed using 50 µL volumes containing: 5 μL 10 x Buffer (containing Mg^2+^), 10 mM of each dNTP, 0.1 mM of each primer, 37.5 µL of ddH _2_O, 2.5 U of Taq DNA Polymerase (Vazyme, China), and 2 µL of DNA template. PCR products were purified with QIAquick PCR purification kit (QIAGEN) following the protocol supplied by the manufacturer. Sequencing was performed by Sangon Biotech (Shanghai, China) on an ABI 3730XL DNA analyzer (Applied Biosystems).

**Table 1:** Information of primers used for PCR programs.

Prime	Sequence
Oph-COI-F	TTTCAACTAATCAYAAGGAYATWGG
Oph-COI-R	CTTCAGGRTGWCCRAARAAYCA
LCO1490	GGTCAACAAATCATAAAGATATTGG
HCO2198	TAAACTTCAGGGTGACCAAAAAATCA

To date, only 10 COI sequences of *Ophioplinthaca* are available from the Genbank and BOLD database (Table 2). In this study, we included another two COI sequences of *O. defensor* from a recent study (Na et al., in press). In total, 19 COI sequences (Table 2), including 5 new sequences and 2 sequences from *Ophiacantha* as outgroup, were used for phylogenetic analysis. COI sequences were aligned using Geneious Prime 2019 with default settings. Phylogenetic analysis was conducted by RAxML (Stamatakis, 2014), with a 1000-replicate bootstrap support value for each node and a GTR+I+G substitution model. The model was selected by the software of jmodeltest– 2.1.10, and the AIC selection results showed the best model was GTR+I+G. Pairwise genetic distance (K2P) were calculated for COI sequences in MEGA6 (Tamura et al., 2013). The Automatic Barcode Gap Discovery (ABGD) analysis (Puillandre et al., 2012) was carried out on the web interface (https://bioinfo.mnhn.fr/abi/public/abgd/abgdweb.html) to establish molecular operational taxonomic units (MOTUs) from COI gene sequence data. The Kimura (K80) model (Kimura, 1980) with a TS/TV of 2.0 (K2P), Pmin = 0.001, Pmax = 0.1, 10 steps and a relative gap width of 1.0.

**Table 2:** COI sequence data used in phylogenetic analysis.

Taxa	Museum registration number	GenBank accession number/BOLD sequence ID
*Ophioplinthaca grandisquama* n. sp.	RSIO56060	MW284982
*Ophioplinthaca grandisquama* n. sp.	RSIO56013	MW284978
*Ophioplinthaca grandisquama* n. sp.	RSIO56014	MW284979
*Ophioplinthaca semele*	RSIO56057	MW284980
*Ophioplinthaca sp.*	RSIO56058	MW284981
Ophioplinthaca pulchra	MV F159608	HM400467
*Ophioplinthaca pulchra*	MV F159607	KU895136
*Ophioplinthaca defensor*	MV F162605	ECHOZ371-10.COI-5P
*Ophioplinthaca defensor*	RSIO410611	MT025802
*Ophioplinthaca defensor*	RSIO410619	MT025808
*Ophioplinthaca globata*	MNHN BP32	KU895134
*Ophioplinthaca rudis*	MNHN BP31	KU895135
*Ophioplinthaca plicata*	MV F144759	EU869990
*Ophioplinthaca plicata*	MV F144758	EU869989
*Ophioplinthaca plicata*	MV F188868	KU895133
*Ophioplinthaca plicata*	MV F144757	ECHOZ372-10.COI-5P
*Ophioplinthaca plicata*	MV F144764	ECHOZ374-10.COI-5P
*Ophiacantha richeri*	NIWA95821	KU895387
*Ophiacantha brachygnatha*	MV F146257	KU895386

**Notes.**

MV Museums Victoria, Australia NIWA National Institute of Water and Atmospheric Research, New Zealand RSIO Second Institute of Oceanology, China

### Nomenclatural acts

The electronic version of this article in Portable Document Format (PDF) will represent a published work according to the International Commission on Zoological Nomenclature (ICZN), and hence the new names contained in the electronic version are effectively published under that Code from the electronic edition alone. This published work and the nomenclatural acts it contains have been registered in ZooBank, the online registration system for the ICZN. The ZooBank LSIDs (Life Science Identifiers) can be resolved and the associated information viewed through any standard web browser by appending the LSID to the prefix http://zoobank.org/. The LSID for this publication is: urn:lsid:zoobank.org:pub:A48B7301-0D4B-4280-BF81-639689F923F6. The online version of this work is archived and available from the following digital repositories: PeerJ, PubMed Central and CLOCKSS.

## Results

### Systematics

**Table:** 

**Figure 2:**
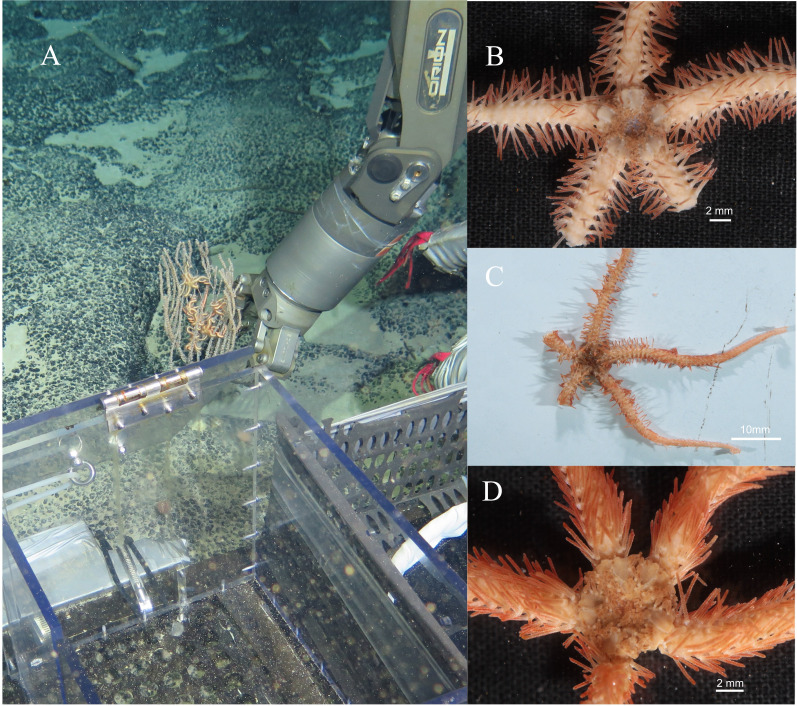
In situ and on board photos of *Ophioplinthaca grandisquama* n. sp. (A) In situ observations, several specimens attached on a Primnoid (*Calyptrophora sp.*). (B–D) Photos on board. (B) Holotype (RSIO56060). (C) Paratype (RSIO56014). (D) Paratype (RSIO56013).

**Figure 3:**
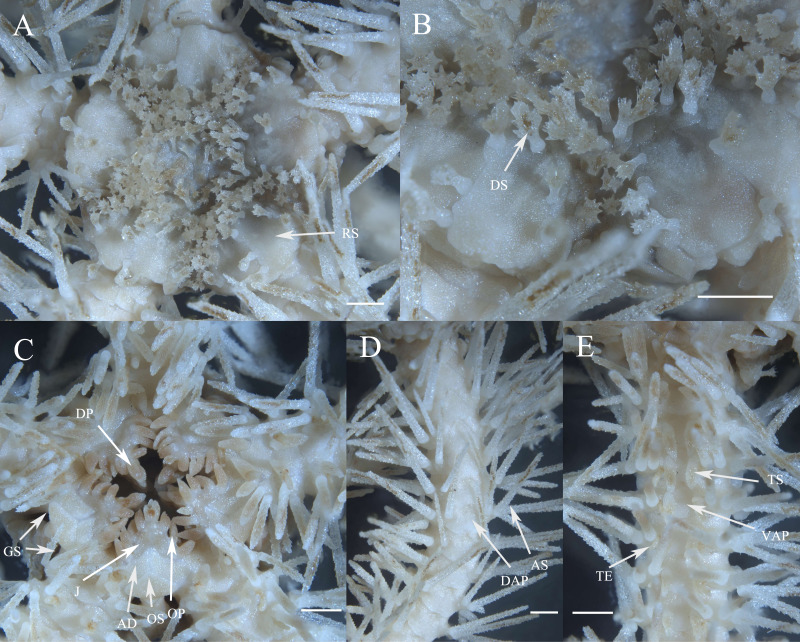
Morphological characters of *Ophioplinthaca grandisquama* n. sp. (Holotype: RSIO56060). (A) Dorsal view of disc. (B) Enlarged disc spines. (C) Ventral view of disc. (D) Dorsal view of arm, proximal part. (E) Ventral view of arm, proximal part. Abbreviations: AD, adoral plate; AS, arm spine; DAP, dorsal arm plate; DP, dental papillae; DS, disc spine; GS, genital slits; J, jaw; OP, lateral oral papilla; OS, oral shield; RS, radial shield; VAP, ventral arm plate; TE, tentacle; TS, tentacle scale. Scale bars: one mm.

**Figure 4:**
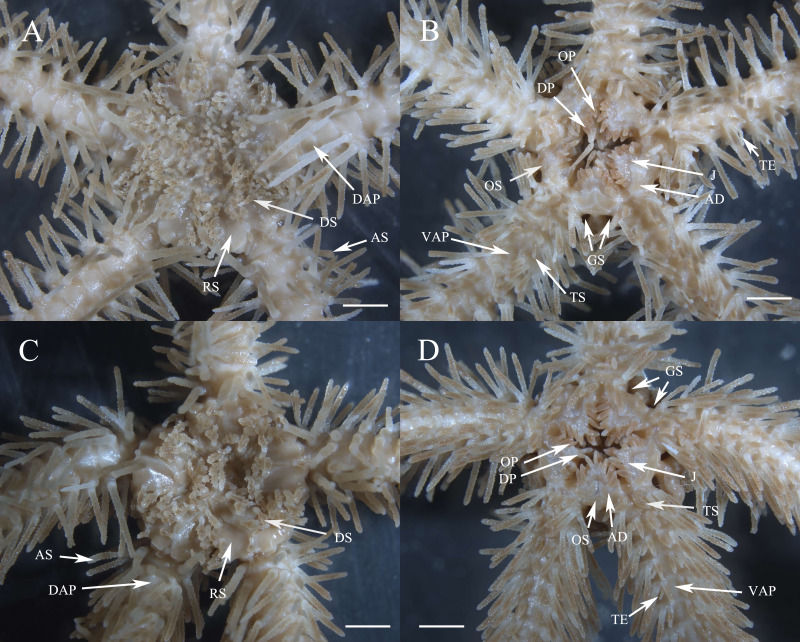
Morphological characters of *Ophioplinthaca grandisquama* n. sp. (Paratype: RSIO56013, RSIO56014). (A–B) Morphological characters of paratype RSIO56014. (A) Dorsal view of disc. (B) Ventral view of disc. (C–D) Morphological characters of paratype RSIO56013. (C) Dorsal view of disc. (D) Ventral view of disc. Abbreviations: AD, adoral plate; AS, arm spine; DAP, dorsal arm plate; DP, dental papillae; DS, disc spine; GS, genital slits; J, jaw; OP, lateral oral papilla; OS, oral shield; RS, radial shield; VAP, ventral arm plate; TE, tentacle; TS, tentacle scale. Scale bars: two mm.

**Figure 5:**
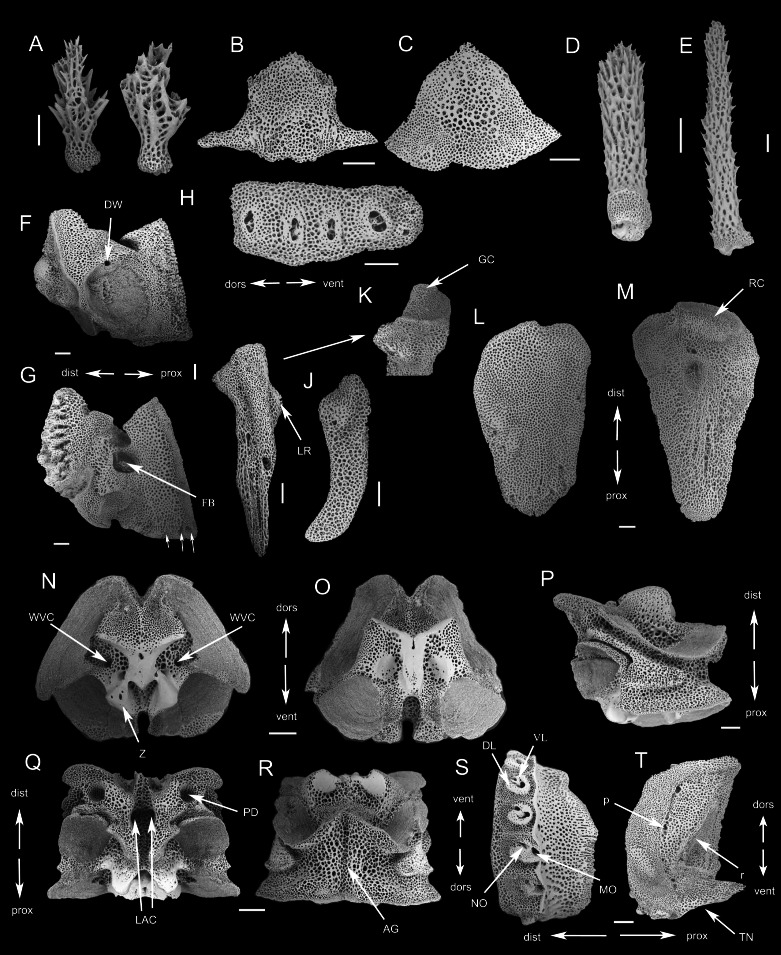
SEM photographs of skeletons of *Ophioplinthaca grandisquama* n. sp. (Paratype: RSIO56014). (A) Disc spine. (B) Ventral arm plate from proximal segment, external view. (C) Dorsal arm plate from proximal segment, external view. (D) Ventral-most arm spine. (E) Dorsal-most arm spine. (F) Oral plate, abradial face. (G) Oral plate, adradial face, white arrows point to oral papillae sockets and pores. (H) Dental plate. (I) Adradial genital plate. (J) Abradial genital plate. (K) Adradial genital plate, distal end. (L) Radial shield, external aspect. (M) Radial shield, internal aspect. (N–R) Vertebrae from proximal portion of arm. (N) Distal view. (O) Proximal view. (P) Lateral view. (Q) Dorsal view. (R) Ventral view. (S) External view of LAP. (T) Internal view of LAP. Abbreviations: AG, aboral groove; DL, dorsal lobe; dors, dorsal; dist, distal; DW, presumable depression for water ring canal; FB, foot basin; GC, adradial genital plate condyle; k, knob; LAC, lateral ambulacral canal; LR, lateral ridge of the adradial genital plate, attachment area of the abradial genital plate; MO, muscle opening; NO, nerve opening; p, perforations; PD, podial basins; prox, proximal; r, ridge; RC, radial shield condyle; TN, tentacle notch; vent, ventral; VL, ventral lobe; WVC, water vascular canal; Z, zagapophyses. Scale bars: 200 µm.

**Class Ophiuroidea Gray, 1840**
**Order Ophiacanthida O’Hara et al., 2017**
**Family Ophiacanthidae Ljungman, 1867**
**Genus*Ophioplinthaca*Verrill, 1899**
***Ophioplinthaca grandisquama*n. sp.** (Figs. 2–5)

urn:lsid:zoobank.org:act:8509E6DB-E902-4A71-9339-EA40725DD688

**Material examined. —** St. RC-ROV05, 161.78°E, 15.54°N, 1049 m, September 17, 2019, 3 specimens (RSIO56013, RSIO56014, RSIO56060).

**Habitat.** All three specimens of the new species were attached to a Primnoid *Calyptrophora sp.* (Fig. 2).

**Etymology.** The specific name alludes to the large and long tentacle scales.

**Description** **of the holotype.** Disc seven mm d.d., high 3.7 mm, five arms, seven times of the disk diameter in length. Disc incised interradially more than 1/5 d.d. creating five wedge-shaped divisions in contrast to the sunken centre and interradii of the disc (Fig. 3A). Each division on aboral surface covered by a pair of large radial shields and a number of irregular plates. Radial shields naked, triangular, about 1/4 d.d. in length, one and a half times as long as wide with a truncate distal edge and a sharp proximal angle, broadly contiguous distally (Fig. 3A). Disc plates overlapping, covered with distinctly elongated disc spines, not enlarged distal to the radial shields interradially. Disc spines stout, up to 0.8 mm in length, 4–6 times as high as wide, bearing numerous distinct thorns on lateral side or apex, some capitate and bifurcated into two prongs at the top, one of the two prongs elongated and inflated (Figs. 3B, 5A). Ventral disc surface covered in small and overlapped plates, few of which bear spines thinner than those on the dorsal surface. Genital slits wide, extending from the oral shields to the dorsal disc surface (Fig. 3C).

Oral shields arrow-head-like shape, with an obtuse proximal angle, rounded laterals and a small obtuse distal lobe, 2 times as wide as long, one of which is expanded as madreporite. Adoral plates quadrilateral, 2 times as long as wide, not separating the oral shields from the lateral arm plate. Jaw triangular, wider than long with 1–2 blunt and serrated dental papillae, and 3–4 conical lateral oral papillae longer than wide with pointed tip, the distal one slightly widened (Fig. 3C). Infradental papilla, adoral plate papillae and lateral oral papillae quite similar in shape so in this study and for descriptive purposes, the ossicles on oral edge of oral plate are all called lateral oral papillae. One oral tentacle scale situated at the end of the jaw slit, slightly larger than oral papillae, often longer than wide with a rounded free edge and covered by distal oral papillae.

Five arms, wide and slightly moniliform. Dorsal arm plates trapezoid to triangular with slightly convex distal edge on proximal segments, contiguous to each other; on distal segments dorsal arm plates change to fan-shaped and just contiguous (Fig. 3D). First ventral arm plates trapezoid much wider than long with a short proximal edge, concave and diverging lateral edges, distal margin much wider. The following plates become pentagonal, slightly wider than long, with a sharp proximal angle, diverging lateral sides which are widely excavated by the corresponding tentacle scales, distal margin board and convex, all separated from each other (Fig. 3E). Tentacle pores covered on the first segments with one or two leaf-shaped scales; one fusiform or conical tentacle scale from the second segments, elongated and thorny with a thick base tapering into a blunt point, slightly longer than one arm segment (Figs. 3C, 3E). Arm spines seven, up to three arm segments in length on proximal arm segments, dorsally four arm spines are thin with distinct lateral thorns, tapering into a sharp point, the second dorsal-most arm spines longest; ventral arm spines shorter and blunt, finely rugose (Fig. 3D). Color in life orange-brown.

**Description of paratypes.** The two paratypes (RSIO56013, RSIO56014) share the same morphological characteristics with the holotype (Figs. 4A–4D, 5A–5E). For one of the two paratypes (RSIO56014) (Figs. 4A, Fig. 4B), the oral structure is incomplete with one of five oral plate sets is missing, which may be due to the malformation or predation. The remaining oral shields are relatively smaller than the holotype, adoral plates are wider. The other paratype (RSIO56013) with stronger disc spines, elongate to flaring head bearing numerous distinct thorns, up to 1.4 mm in length, 4–6 times as high as wide (Figs. 4C, 4D). Tentacle pores covered, on the proximal arm segments one elongated scale with a rounded base tapering to a blunt point.

**Description of the skeletal elements** (Paratype: RSIO56014): Oral plates longer than high, with a small pore for water ring canals, abradial muscle fossa large with finer mesh stereom than remaining ossicle (Fig. 5F). A row of three papilla sockets and pores near lower edge of adradial proximal oral plate as articulations of oral papillae; conspicuous s-curved suture line crosses foot basin (Fig. 5G). Dental plate entire with single column of wide sockets, with low dorsal and ventral border, not penetrating (Fig. 5H). Adradial genital plate long, articulation surface with slightly elevated elongated condyle (Fig. 5I, 5K); abradial genital plate slightly smaller in size than adradial plate and articulating proximal to the adradial plate condyle (Fig. 5J). Radial shield longer than wide, with abradial projection and convex radial edge (Fig. 5L). Internally, radial shield with one distal domed condyle and one depression, which articulated with genital plate (Fig. 5M).

The vertebrae articulation zygospondylous, wider than long in proximal segments and gradually changes to longer than wide from the middle to distal segments, with zygapophyses framing the water vascular canal on proximal side (Figs. 5N–5R). A longitudinal groove on oral side (Fig. 5Q), with a pair of lateral ambulacral canals opening inside the oral groove (Fig. 5Q); the podial basins on the oral side are small, 127 µm in diameter (Fig. 5Q); an aboral groove on the aboral side is slightly expressed without extension (Fig. 5R). Lateral arm plates (LAP) with constriction in proximal part leading to raised distal portion (Fig. 5S). The external surface of the LAP consisted of regularly meshed stereom, mesh size gradually decreasing from the middle to the proximal margin, while in the distal part, mesh size is mostly small but larger near the distal margin (Fig. 5S). Arm spine articulations well developed, volute-shaped, dorsal and ventral lobes merged at their proximal tips, sigmoidal fold present (Fig. 5S). The muscle opening is larger than the nerve opening (Fig. 5S). On the internal side are a row of perforations on the central of middle part, parallel to the arrangement of spine articulation (Fig. 5T); a continuous ridge and a prominent knob close to the ventral edge forming vertebral articular structures, of which shape is reminiscent of an undivided digit 1 with a broad, nose-shaped beak (Fig. 5T).

**Remark.** *Ophioplinthaca grandisquama* n. sp**.** is characterized by the stout disc spines, capitate with typically elongate to flaring head bearing numerous distinct thorns, radial shields roughly triangular, about 1/4 d.d. in length and contiguous distally, the tentacle scales elongated and stout. The thick tentacle scales in *O. grandisquama* n. sp**.** are elongated with a rounded base tapering to a blunt point and covered in irregular thorns similar to arm spines, which is distinctly distinguished from its congeners and most of them bearing oval or leaf-shaped tentacle scales (Thomson, 1877; Lyman, 1878; Lyman, 1883; Clark, 1900; Koehler, 1904; Clark, 1911; Koehler, 1922; Mortensen, 1933; Clark, 1939; Koehler, 1930; Clark, 1949; John & Clark, 1954; Cherbonnier & Sibuet, 1972; Guille, 1981; O’Hara & Stöhr, 2006; Table 3).

**Table 3:** Comparison of of key morphological characters among species from the genus *Ophioplinthaca*, based on literature.

Species	Disc spines	Size of radial shields	Position of radial shields	Shape of tentacle scale	Reference
*Ophioplinthaca abyssalis*	elongate and conical granules, smooth	1/3 d.d., 3 times as long as wide	contiguous or just separate distally, sunken	conical and pointed	Cherbonnier & Sibuet (1972)
*Ophioplinthaca amezianeae*	tall and slender spines, rounded base terminating in 2–3 small thorns	1/6 d.d., 2 times as long as wide	Separated	long and spiniform	O’Hara & Stöhr (2006)
*Ophioplinthaca athena*	elongate conical granules, with a few radiating spinules at the end	1/3 d.d., 4 times as long as wide	contiguous distally	oval to slender	Clark (1949)
*Ophioplinthaca bythiaspis*	spherical to conical to cylindrical granules, with a few small terminal thorns	1/3–1/4 d.d., 4 times as long as wide	separated, sunken	oval to bottle-shaped	Clark (1911) and O’Hara & Stöhr (2006)
*Ophioplinthaca carduus*	small cylindrical granules, with a crown of thorns at the end	1/4 d.d., 2 times as long as wide	Separated	conical and pointed, with one or more side thorns	Lyman (1878)
*Ophioplinthaca chelys*	short and blunt stumps, usually smooth, which also present over each arm	1/3–1/4 d.d., 4-5 times as long as wide	Separated, deeply sunken	thick and pointed, flattened, sensibly smooth	Thomson (1877)
*Ophioplinthaca citata*	small and cylindrical stumps, with a terminal crown of thorns	1/4 d.d., 3–4 times as long as wide	separated, sunken	oval to elliptical	Koehler (1904) and O’Hara & Stöhr (2006)
*Ophioplinthaca clothilde*	cylindrical stumps, terminating in a flaring irregular crown of a dozen or more spinnles	1/6 d.d., 2 times as long as wide	contiguous in the outer fourth	narrow and sharply pointed, with numerous prickles about its tip.	Clark (1949)
*Ophioplinthaca codonomorpha*	minute and rough granules	1/6–1/8 d.d., 1.5 times as long as wide	Separated	oval to pointed	Clark (1911)
Ophioplinthaca crassa	low and cylindrical granules	1/6 d.d., 1-1.5 times as long as wide	Separate or just contiguous distally	slender and pointed	Clark (1939)
*Ophioplinthaca defensor*	round to cylindrical granules, nearly smooth	1/2–1/3 d.d., two times as long as wide	contiguous on almost all the length	rounded or oval	Koehler (1930) and Na et al. (in press)
*Ophioplinthaca dipsacos*	minute stumps with a crown of thorns at the top, which also present over each arm	1/4 d.d., 2 times as long as wide	contiguous or just separate distally	elongated and pointed, with one or two microscopic thorns	Lyman (1878)
*Ophioplinthaca globata*	cylindrical to conical stumps, with obvious thorns at the upper half	1/5–1/8 d.d., 1-2 times as long as wide	contiguous distally or completely separated	oval	Koehler (1922); O’Hara & Stöhr (2006)
*Ophioplinthaca grenadensis*	strong glassy spines, thick at the base but rapidly taper, with smaller spines on all sides and ending in two or three thorns.	1/6–1/8 d.d., 1.5 times as long as wide	separated	large and leaf-like, pointed	John & Clark (1954)
*Ophioplinthaca hastata*	stout and capitate stumps, with a convex to flaring head bearing numerous small thorns	1/6 d.d., 1.5 times as long as wide	contiguous or just separate distally.	clavate, terminally spiniform	Koehler (1922); O’Hara & Stöhr (2006)
*Ophioplinthaca incisa*	conical to cylindrical stumps, smooth	1/4 d.d., 2 times as long as wide	contiguous distally, a little sunken	oval and thickened	Lyman (1883)
*Ophioplinthaca laudator*	thin and elongated stumps, with four to five divergent and pointed thorns at the top	1/4–1/6 d.d., 2 times as long as wide	contiguous distally, a little sunken	elongated and pointed	Koehler (1930)
* Ophioplinthaca lithosora*	low and cylindrical stump, with two to six small thorns near the top	1/4 d.d., 3 times as long as wide	Separated, a little sunken	long and rounded at tip or pointed	Clark (1911)
*Ophioplinthaca manillae*	elongated and cylindrical stump, terminated by several sharp points forming a crown, or divided into three digits	1/4–1/6 d.d., as long as wide	contiguous distal half	pointed and elongate, more strongly denticulate	Guille (1981)
*Ophioplinthaca miranda*	rounded granules, base narrows in a very short pedicle, trimmed with fine pointed asperities	1/6 d.d., 2 times as long as wide	contiguous in the outer fourth	small and oval	Koehler (1904)
Ophioplinthaca monitor	short and bowl-shaped stumps, with an expanded apex covered in sharp thorns	1/4 d.d., 2.5 times longer	widely separated, sunken	oval to spongy	Koehler (1930); O’Hara & Stöhr (2006)
*Ophioplinthaca papillosa*	elongated and cylindrical stump, terminated by 3-6 thorns	1/3 d.d., 2 times as long as wide	broadly contiguous	flat and pointed	Clark (1939)
*Ophioplinthaca plicata*	conical to cylindrical to capitate granules, finely rugose or rarely with a few thorns	1/3–1/4 d.d., 2-2.5 times as long as wide	contiguous distally	erect, curved inwards with a pointed to rounded tip	Lyman (1878); O’Hara & Stöhr (2006)
*Ophioplinthaca pulchra*	spherical to capitate stumps, nearly smooth	1/3 d.d., 2–2.5 times as long as wide	Separate or contiguous distally	Small and conical	Koehler (1904); O’Hara & Stöhr (2006)
*Ophioplinthaca rudis*	long and slender spines, needle-like, smooth to finely serrate	1/3 d.d., 1–2 times as long as wide	Separate or contiguous distally	bottle-shaped to pointed	Koehler (1897) and O’Hara & Stöhr (2006)
*Ophioplinthaca sarsii*	short and stout stump, smooth, which also present over each arm	2 times as long as wide	widely separated, sunken	stout and pointed, flattened, cloven or jagged on the edges	Lyman (1878)
*Ophioplinthaca semele*	thick and swollen cylindrical stumps, with a few short and flaring thorns at the top	more than 1/4 d.d., 2.5-3 times as long as wide	contiguous in the outer third	spinous, more pointed	Clark (1949)
*phioplinthaca sexradia*	conical granules, smooth	1/2 d.d., 2-3 times as long as wide	contiguous distally	small and oval	Mortensen (1933)
*Ophioplinthaca spinissima*	small and thorny stumps	1/3–1/4 d.d., 2 times as long as wide		large and pointed	Clark (1900)
*Ophioplinthaca tylota*	knob-like tubercle, typically bud-like with a short stalk which merges into the ellipsoid tubercle itself	1/4 d.d., 3 times as long as wide	contact and overlaps	flat and pointed, thorny	Clark (1939)
*Ophioplinthaca weberi*	no	1/3 d.d., 2 times as long as wide	contiguous in the outer half	small and oval	Koehler (1904)
* Ophioplinthaca grandisquama* n. sp.	long and stout spines, capitate with typically elongate to flaring head bearing numerous distinct thorns	1/4 d.d., 1.5 times as long as wide	contiguous distally	long and thorny, with a trunk base tapering into a blunt point	Present study

The sizes and shapes of radial shields and disc spines have been suggested to be the primary criteria for delimiting species (O’Hara & Stöhr, 2006). We compared the key morphological characters among species from the genus *Ophioplinthaca* (Table 3). *O. hastata* Koehler, 1922 and *O. globata* Koehler, 1922, which resemble the new species mostly, also have stout and capitate disc spines. However, in *O. grandisquama* n. sp**.,** the disc spines are more elongated, 4-6 times as high as wide, bearing numerous distinct thorns all over the whole spine except the basal trunk, whereas the disc spines are only 2–3 times as high as wide, capitate with a convex to flaring head bearing numerous small thorns in *O. hastata*, and are cylindrical to conical with obvious thorns only in the upper half in *O. globata* with similar height-width ratio to *O. hastata* (O’Hara & Stöhr, 2006). Radial shields are relatively small, in *O. hastata* and *O. globata*, with 1/6 d.d. and 1/5 to 1/8 d.d. in length, respectively, and contiguous distally or separate, instead of 1/4 d.d. in length, broadly contiguous distally in the new species. Additionally, dorsal arm plates are also different between the new species and *O. hastata* and *O. globata*. Dorsal arm plates are a little longer than wide or as wide as long, separated from the basal arm segments, instead of contiguous at least on proximal segments in *O. grandisquama* n. sp.

Other species, *O. ameziane*ae O’Hara & Stöhr, 2006 and *O. rudis* (Koehler, 1897), were described with elongated spines, greater than 3 times as high as wide in this genus. The former is clearly different from *O.grandisquama* n. sp. in having slender disc spines, with a rounded base tapering to a sharp point or terminating in 2–3 small thorns, radial shields separate, oral shields as long as wide. The latter can be distinguished by having needle-like disc spines, long and slender, up to 1.3 mm in length, smooth to finely serrate, pentagonal oral shields, and bottle-shaped to pointed tentacle scales, half as long as the ventral arm plate.

**Table:** 

**Figure 6:**
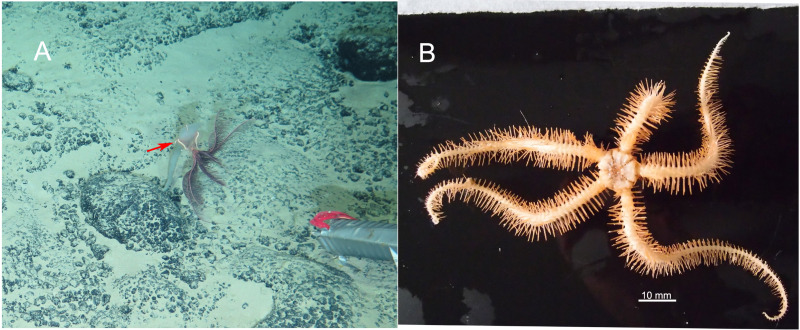
In situ (A) and on board (B) photos of *Ophioplinthaca semele*.

**Figure 7:**
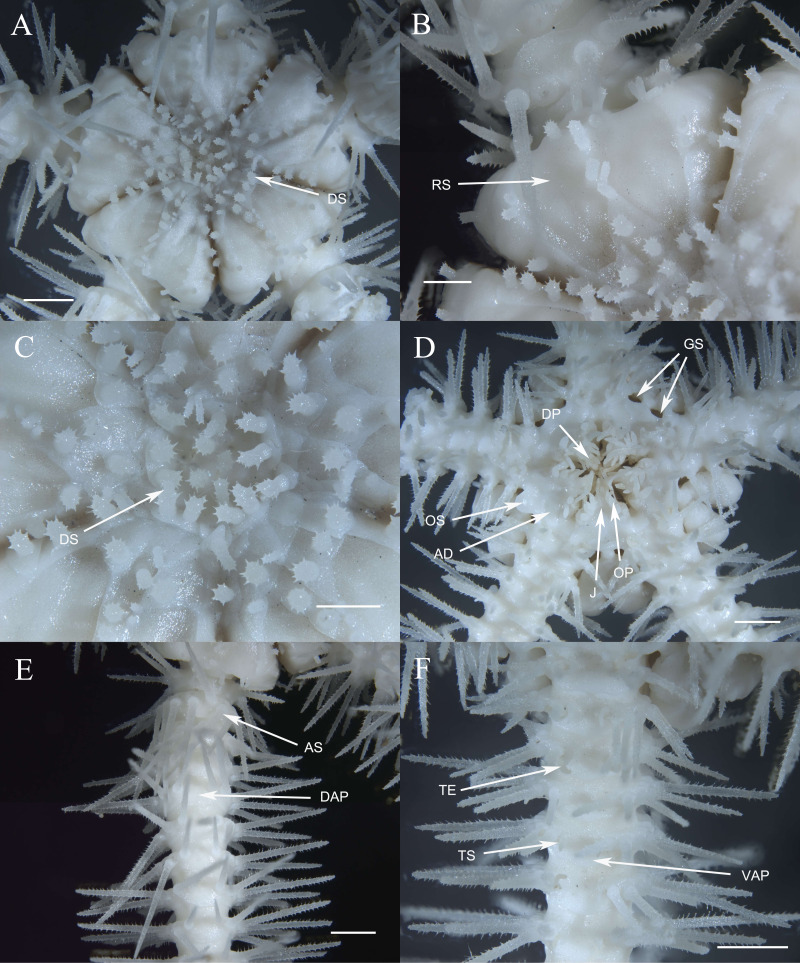
Morphological characters of *Ophioplinthaca semele* (RSIO56057). (A) Dorsal view of disc. (B) Radial shields. (C) Disc spines. (D) Ventral view of disc. (E) Dorsal view of arm, Proximal part. (F) Ventral view of arm, proximal part. Abbreviations: AD, adoral plate; AS, arm spine; DAP, dorsal arm plate; DP, dental papillae; DS, disc spine; GS, genital slits; J, jaw; OP, lateral oral papilla; OS, oral shield; RS, radial shield; VAP, ventral arm plate; TE, tentacle; TS, tentacle scale. Scale bars: one mm (B, C), two mm (A, D–F).

**Figure 8:**
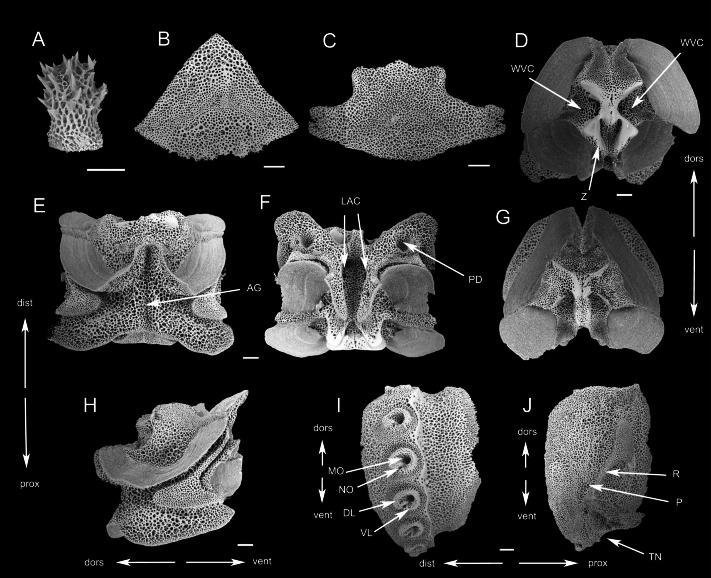
SEM photographs of *Ophioplinthaca semele* (RSIO56057). (A) Disc spine. (B) Dorsal arm plate from proximal segment, External view. (C) Ventral arm plate from proximal segment, external view. (D–H) Vertebrae from proximal portion of arm. (D) distal view. (E) Dorsal view. (F) Ventral view. (G) Proximal view. (H) Lateral view. (I) External view of lateral arm plate. (J) Internal view of lateral arm plate. Abbreviations: AG, aboral groove; DL, dorsal lobe; dors, dorsal; dist, distal; LAC, lateral ambulacral canal; MO, muscle opening; NO, nerve opening; P, perforations; PD, podial basins; prox, proximal; R, ridge; TN, tentacle notch; vent, ventral; VL, ventral lobe; WVC, water vascular canal; Z, zagapophyses. Scale bars: 200 µm.

***Ophioplinthaca semele*(Clark, 1949)** (Figs. 6–8)

**Material examined. —** St. RC-ROV08, 161.81°E, 15.53°N, 1024 m, September 20, 2019, 1 specimen (RSIO56057).

**Habitat and Distribution.** This specimen was found attaching on a blade-like glass sponge together with a sea lilly (Fig. 6). The holotype and other specimens were collected near Hawaii (537–1,250 m); this is the first record of this species from a seamount in the Northwest Pacific (1,024 m).

**Description of morphological characteristics.** Disc 11.2 mm d.d., high 5.4 mm, five arms, seven times of the disk diameter in length. Disc almost incised interradially 1/3 d.d., creating five wedge-shaped divisions covered by a pair of large, naked radial shields and a number of irregular plates (Fig. 7A). Radial shields triangular, about 1/3 d.d. in length, 1.5–2 times as long as wide with a truncate distal edge and blunt proximal angles, contiguous for 1/3–1/2 of the length (Fig. 7B). Disc plates overlapping, bearing cylindrical swollen stumps, up to 0.5 mm high, covered in obvious thorns on the upper half (Figs. 7C, 8A). Disc spines at the distal margin and between radial shields are thinner with less thorns (Fig. 7C). Ventral disc surface covered in small overlapping plates, without spines. Genital slits long and wide (Fig. 7D).

Oral shields diamond-shaped, with an obtuse proximal angle, rounded laterals and an obtuse to lobed distal angle, 2 times as wide as long, one of which expanded as madreporite (Fig. 7D). Adoral plates quadrilateral, large and broad, two times as long as wide, not separating the oral shields from the first lateral arm plate. Jaw triangular, as long as wide with 2–3 thin and long dental papillae. Lateral oral papillae 4–5, pointed, up to 3 times longer than wide, and the distal two oral papillae slightly broadened and leaf-shaped, standing erect, abutting the elongated oral tentacle scale (Fig. 7D).

Five arms, wide and slightly moniliform. First dorsal arm plate wider than long with obtuse proximal angle and straight distal border. Succeeding plates triangular to scallop-shaped with convex distal edge, slightly wider than long, separated from each other (Figs. 7B, 7E, 8B). Ventral arm plates pentagonal with a sharp proximal angle, diverging lateral sides which are very widely excavated by the corresponding tentacle scales, distal side convex, widely separated from each other (Figs. 7F, 8C). Tentacle pores on the first arm segments, covered with one or two scales, leaf-like, pointed and spiniform, more than half length of ventral arm plates, decreasing to one scale thereafter until nearly the end of the arm (Figs. 7D, 7F). The proximal arm segments bearing up to eight arm spines, with sharp tip and distinct teeth, almost meeting each other on the dorsal mid-line on the fourth segment (Fig. 7E). The third dorsalmost arm spines are the longest, up to three segments in length, lowermost shortest, one segment in length. As the arm segments reduced distally, arm spines reduced to five. Color in life orange-white.

**Description of the skeletal elements**. The vertebrae articulation zygospondylous, wider than long in proximal segments, gradually changing to longer than wide from the middle to distal segments, with zagapophyses framing the water vascular canal on proximal side (Figs. 8D–8H). The aboral groove on the dorsal side is moderately expressed without extension (Fig. 8E); a longitudinal groove on oral side (Fig. 8F), with a pair of lateral ambulacral canals opening inside the oral groove (Fig. 8F); the podial basins on the oral side are small (Fig. 8F). LAP with constriction in proximal part leading to raised distal portion. Arm spine articulations well developed, volute-shaped, dorsal and ventral lobes merged at their proximal tips, sigmoidal fold present (Fig. 8I). The muscle opening is larger than the nerve opening. On the internal side, a group of small, irregular perforations parallel to the arrangement of spine articulation; a continuous ridge and a prominent knob forming vertebral articular structures, of which shape is reminiscent of an undivided digit one with a broad, nose-shaped beak (Fig. 8J).

**Remark.** This specimen was identified as *O. semele* (Clark, 1949) based on the multiple apical papillae, large radial shields contiguous for 1/3–1/2 of the length distally, cylindrical disc stumps with obvious thorns on the top and upper half. It also has some slight differences, having two or three shaped and leaf-shaped tentacle scales on the first tentacle pore instead of three or more broad and spoon-shaped scales in the holotype, adoral plates complete instead of divided into two or more plates in the holotype. Clark (1949) described the differences in tentacle scales and lateral oral papillae between the two smaller specimens and the holotype. Furthermore, tentacle scale morphology is not reliable for species delimitating in the genus *Ophioplinthaca* (O’Hara & Stöhr, 2006). Therefore, with only one specimen of this species in our collection, these differences are attributed to inter-species variation rather than characteristics for taxonomic delimitation.

The cylindrical disc granules with a flaring top of a few thorns are reminiscent of *Ophioplinthaca citata* Koehler, 1904 from the New Caledonia, which differed in having narrower radial shields, contiguous dorsal and ventral arm plates and single ventral-most teeth (O’Hara & Stöhr, 2006). Several other species are also close to *O. semele* in the shape of disc spines. *Ophioplinthaca globata* also has cylindrical to conical granules, the upper half covered in obvious thorns, but can be differentiated in having a single ventral-most tooth, and some other differences such as size and shape of radial shields and jaws, and number of arm spines (Koehler, 1922; O’Hara & Stöhr, 2006). *Ophioplinthaca clothilde* (Clark, 1949) has stumps terminating in flaring irregular crown of a dozen or more spines, and *O. lithosora* (Clark, 1911) has low cylindrical stumps with two to six tiny thorns near the apex. But they can be distinctly distinguished by size of radial shields and the number of apical papillae.

**Table:** 

**Figure 9:**
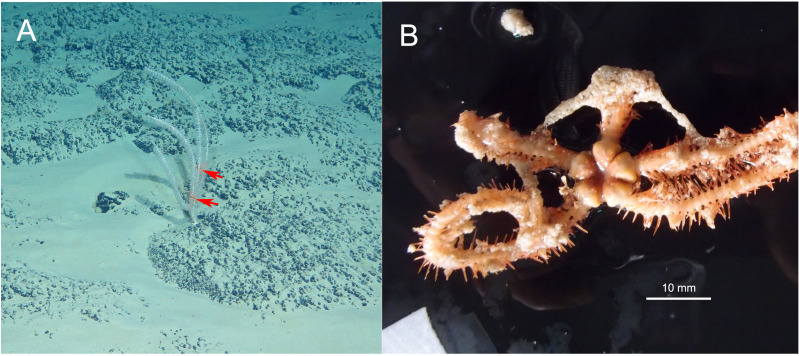
In situ (A) and on board (B) photos of *Ophioplinthaca* sp.

**Figure 10:**
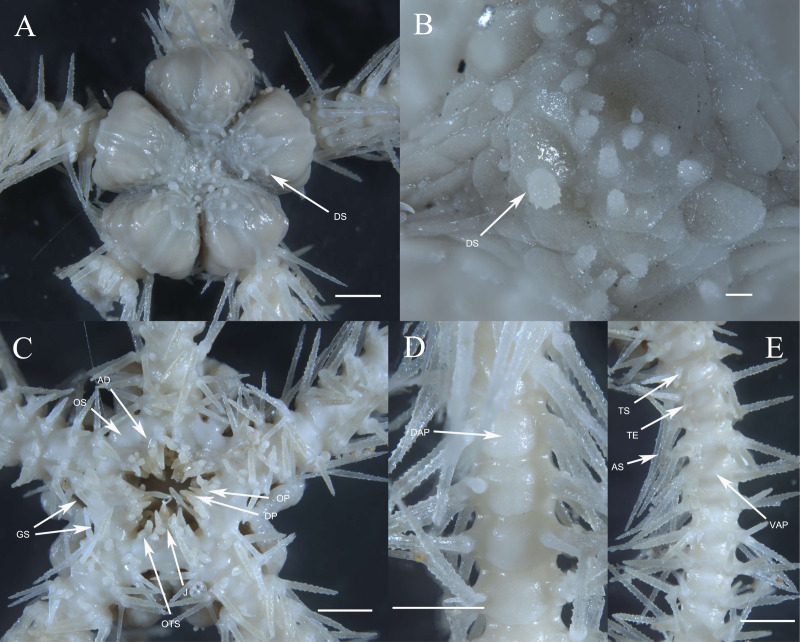
Morphological characters of *Ophioplinthaca* sp. (RSIO56058). (A) Dorsal view of disc. (B) Disc spines. (C) Ventral view of disc. (D) Dorsal view of arm, proximal part. (E) Ventral view of arm, proximal part. Abbreviations: AD, adoral plate; DP, dental papillae; AS, arm spine; DAP, dorsal arm plate; DS, disc spine; GS, genital slits; J, jaw; OP, lateral oral papilla; OS, oral shield; OTS, oral tentacle scale; RS, radial shield; VAP, ventral arm plate; TE, tentacle; TS, tentacle scale. Scale bars: two mm (A, C–E), 0.2 mm (B).

**Figure 11:**
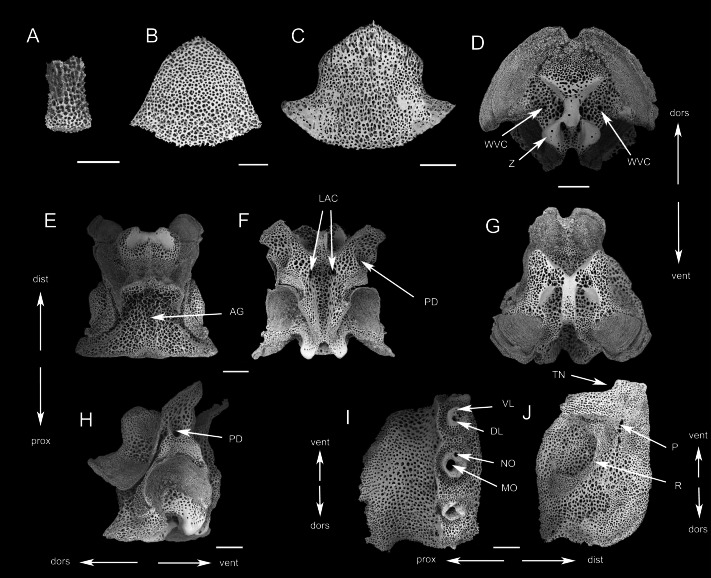
SEM photographs of *Ophioplinthaca* sp. (RSIO56058). (A) Disc spine. (B) Dorsal arm plate from proximal segment, external view. (C) Ventral arm plate from proximal segment, external view. (D–H) Vertebrae from proximal portion of arm. (D) proximal view. (E) Dorsal view. (F) Ventral view. (G) Distal view. (H) Lateral view. (I) External view of lateral arm plate. (J) Internal view of lateral arm plate. Abbreviations: AG, aboral groove; DL, dorsal lobe; dors, dorsal; dist, distal; LAC, lateral ambulacral canal; MO, muscle opening; NO, nerve opening; P, perforations; PD, podial basins; prox, proximal; R, ridge; TN, tentacle notch; vent, ventral; VL, ventral lobe; WVC, water vascular canal; Z, zagapophyses. Scale bars: 200 µm.

***Ophioplinthaca*sp.** (Figs. 9–11)

**Material examined. —** St. RC-ROV08, 161.80°E, 15.52°N, 1,146 m, September 20, 2019, 1 specimen (RSIO56058).

**Habitat.** This specimen was found attaching on a *Narella* (Fig. 9).

**Description of morphological characteristics.** Disc 10.4 mm d.d., high 4.2 mm, arms seven times d.d.. Disc incised interradially 1/3 d.d., creating a wedge over each arm base, wedges tumid, in contrast to the sunken centre and interradii of disc (Fig. 10A). Radial shields naked, triangular, more than 1/4 d.d. in length, 1.5∼2 times as long as wide with a round distal margin and a sharp proximal angle, distally contiguous more than half of the length, and separated proximally by a triangular plate (Fig. 10A). The center of the disc is occupied by small irregular plates, bearing small granules up to 0.25 mm high, 1∼1.5 times as high as wide, cylindrical to capitate with a terminal crown of thorns (Figs. 10B, 11A). Ventral disc surface covered in small and uneven plates without granules (Fig. 10C). Genital slits long and wide.

Oral shields diamond-shaped, with an obtuse proximal angle, rounded laterals and an obtuse to lobed distal angle, 2 times as wide as long, one of which is expanded into a madreporite (Fig. 10C). Adoral plates quadrilateral, 3 times as long as wide, not separating the oral shields from the first lateral arm plate. Jaw triangular, wider than long with 1 blunt dental papilla, and 3 lateral oral papillae that are swollen and conical, gradually decreasing in size from inside to outside. One large oral tentacle scale situated under the distal oral papillae, conical and elongate, up to two mm long (Fig. 10C).

Five arms, wide and slightly moniliform. Dorsal arm plates triangular to scallop-shaped with convex distal edge, separated from each other (Figs. 10D, 11B). Ventral arm plates pentagonal, separated from each other, with a small proximal angle, diverging lateral sides which are excavated by the corresponding tentacle pores, and distal side convex (Figs. 10C, 10E, 11C). Tentacle pores on the first arm segments, covered with one or two scales, decreasing to one scale thereafter to the end of the arm. Tentacle scales thick and smooth on the basal segments, change to smaller, leaf-like and thorny on the following segments, almost half length of the ventral arm plates (Figs. 10C, 10E). The proximal arm segments with up to seven spines, of which the dorsally second or third are the longest, three segments in length, lowermost shortest, one segment in length (Fig. 10D). The number of arm spines reduced to four on distal segments.

**Description of the skeletal elements**. The vertebrae articulation zygospondylous, wider than long in proximal segments and gradually changing to longer than wide on middle to distal segments, with zagapophyses framing the water vascular canal on proximal side (Figs. 11D–11H). The aboral groove on the dorsal side is moderately expressed without extension (Fig. 11E); a longitudinal groove on oral side (Fig. 11F), with a pair of lateral ambulacral canals opening inside the oral groove (Fig. 11F); the podial basins on the oral side are small (Fig. 11F, 11F). LAPs with constriction in proximal part leading to raised distal portion (Fig. 11I). Arm spine articulations well developed, volute-shaped, dorsal and ventral lobes merged at their proximal tips, sigmoidal fold present (Fig. 11I). The muscle opening is larger than the nerve opening (Fig. 11I). On the internal side, a group of small, irregular perforations parallel to the arrangement of spine articulation; a continuous ridge and a prominent knob forming vertebral articular structures, of which shape is reminiscent of an undivided digit one with a broad, nose-shaped beak (Fig. 11J).

**Remark.** This specimen is characterized by the deep interradial incisions, radial shields twice as long as wide, 1/4 d.d. in length, contiguous for most of their length, the disc spines cylindrical to capitate with a terminal crown of thorns, and jaw wider than long with 1 blunt dental papilla and 3 small lateral oral papillae in each side, gradually decreasing in size from inside to outside. *Ophioplinthaca pulchra* Koehler, 1904 is similar to our specimen in the shape of disc spines, but it differs in having some spherical and smooth disc granules, large radial shields, up to 1/3 mm d.d., only contiguous distally, oral shields much longer than wide, and four pointed to square-shaped lateral oral papillae in each side. *Ophioplinthaca pulchra* is quite similar to *Ophioplinthaca plicata* (Lyman, 1878), and can be difficult to distinguish*. Ophioplinthaca plicata* is highly variable, particularly in the shape of the disc stumps, the position of the radial shields, and the shape of oral shields (O’Hara & Stöhr, 2006). Some features of this specimen fall within the range of variation, such as the broadly contiguous radial shield and small oral shield, but the capitate disc spines and only three small lateral oral papillae on each side of jaws can be distinguished from *O.plicata*. However, the limits of species in genus *Ophioplinthaca* are obscure (O’Hara & Stöhr, 2006) and with only one specimen, it is impossible to provide a full description of the range of variation and stable characteristics for diagnosis, therefore, we prefer not to attach a name to this single specimen.

### Lateral arm plate characteristics

Lateral arm plates (LAPs) have been suggested to the key taxonomic character for Ophiuroidea (Martynov, 2010), and are potentially identifiable to species level (Thuy & Stöhr, 2011; Thuy & Stöhr, 2016). Spine articulations of LAPs have been amply studied in recent systematic studies (Thuy & Stöhr, 2011; Thuy & Stöhr, 2016; Stöhr, O’Hara & Thuy, 2012; Thuy, 2013; O’Hara et al., 2018). However, recent research suggested that some of species displayed indistinguishable lateral arm plate morphologies, but belonged to the same genus in all cases (Thuy & Stöhr, 2011). Our study endorses the use of vertebral articular structures for taxonomic interpretations, including species and genus identifications, providing that descriptions are based on pristinely preserved proximal LAPs.

**Figure 12:**
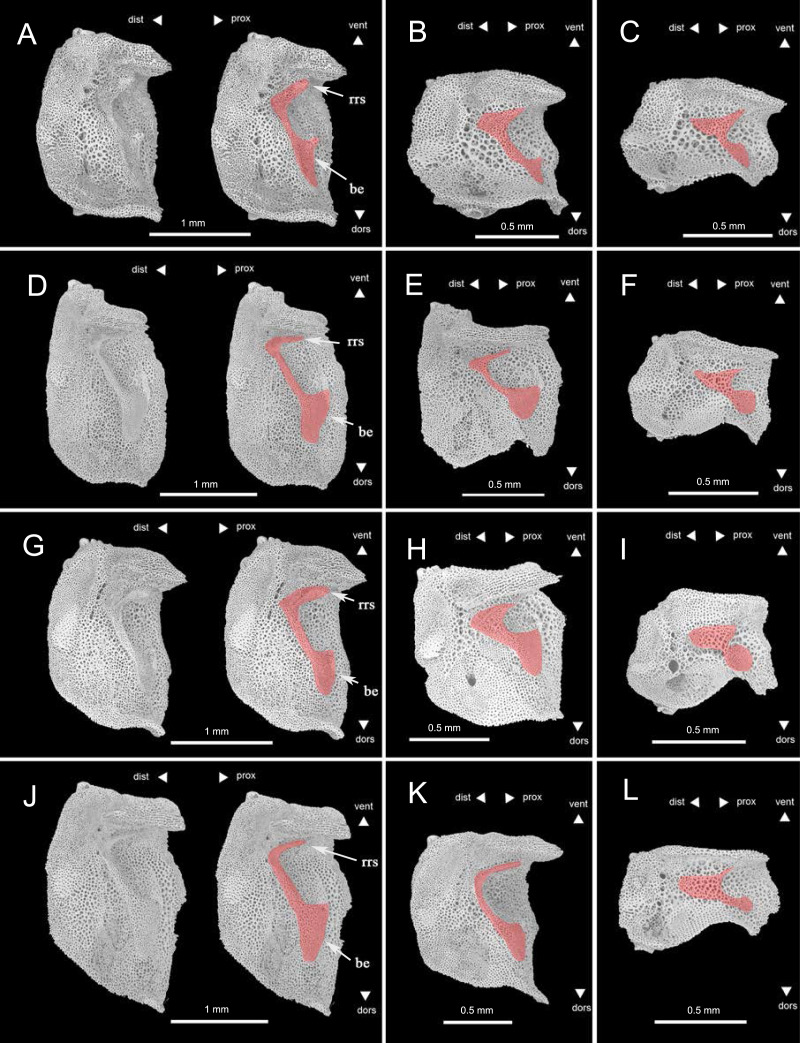
Lateral arm plates of four species of *Ophioplinthaca* from the proximal to distal segments of the arm, all shown with ventral edges upwards (in order to compare with existing research, refer to the layout format of Numberger-Thuy & Thuy (2020). (A–C) *Ophioplinthaca grandisquama* n. sp., (A) Proximal arm segments, (B) Middle arm segments, (C), distal arm segments; (D–F) *Ophioplinthaca semele*, (D) proximal arm segments, (E) middle arm segments, (F), distal arm segments; (G–I) *Ophioplinthaca sp*., (G) proximal arm segments, (H) middle arm segments, (I), distal arm segments; (J–L) *Ophioplinthaca defensor*, (J) proximal arm segments, (K) middle arm segments, (L), distal arm segments. The vertebral articular structures marked in red, like an undivided digit 1 with a broad, nose-shaped beak. Abbreviations: be, beak; rrs, right root serif.

There have been few studies on the ridges and knobs (vertebral articular structures) on the inner side of the lateral arm plates, in contrast, although they were recently confirmed to be diagnostic on various taxonomic levels (Thuy & Stöhr, 2011; Thuy & Stöhr, 2016; Stöhr, O’Hara & Thuy, 2012; Thuy, 2013; Numberger-Thuy & Thuy, 2020). Numberger-Thuy & Thuy (2020) introduced ‘vertebral articular structures of the lateral arm plate’ as an anatomically consistent term to designate all ridges, knobs and other structures on the inner side of the lateral arm plate, and examined the shape of vertebral articular structures of several Ophiacanthid species. Among which, the vertebral articular structures of species *Ophioplinthaca plicata* was similar to *Ophiacantha serrata* which has close relationship with the genus *Ophioplinthaca*, like an undivided digit 1 with a broad, nose-shaped beak. To test whether LAP is useful in distinguishing species in the genus *Ophioplinthaca*, LAPs of the three species described in this study and *O. defensor* reported by Na et al. (in press) were compared (Fig. 12). The shape of vertebral articular structures of the four *Ophioplinthaca* species are consistent with *O. plicata* (Numberger-Thuy & Thuy, 2020), an undivided digit one with a broad, nose-shaped beak, supporting the monophyly of the genus *Ophioplinthaca*. The shape of vertebral articular structures changed from the proximal to distal segments, and the “beak” on the proximal edges gradually shrank but not divided in the mid- or distal segments (Fig. 12). Although vertebral articular structures were similar among *Ophioplinthaca* species, there were is slight differences between the four species, in the shape of the undivided digit “1” and the nose-like ”beak”. In the new species, the “beak” was more like hook nose, with two sharp acute angles proximally (Fig. 12A). For the other three species herein, the “beaks” were distinctly broadened compared to the new species. For the *O. semele*, the “beak” curved proximal-ward with an extremely blunt angle dorsally. For *O. defensor* and *O*. sp., the “beaks” were close to that in *O. plicata* (Numberger-Thuy & Thuy, 2020), in righttriangle shape, but slightly curved proximal-ward (Figs. 12G, 12J). Furthermore, the right root serif was slender in *O. semele* and *O. defensor*, similar to *O. plicata*, but stouter in *O. grandisquama* n. sp. and *O*. sp. In general, the vertebral articular structure seems to be a potentially useful characteristic for identification at species level, especially for the genus *Ophioplinthaca*, which is morphologically confusing among species. However, with only limited material examined, it’s still difficult make any conclusion. An exhaustive investigation on lateral arm plates is required to assess these characteristics for taxonomic identification.

### Phylogeny

**Figure 13:**
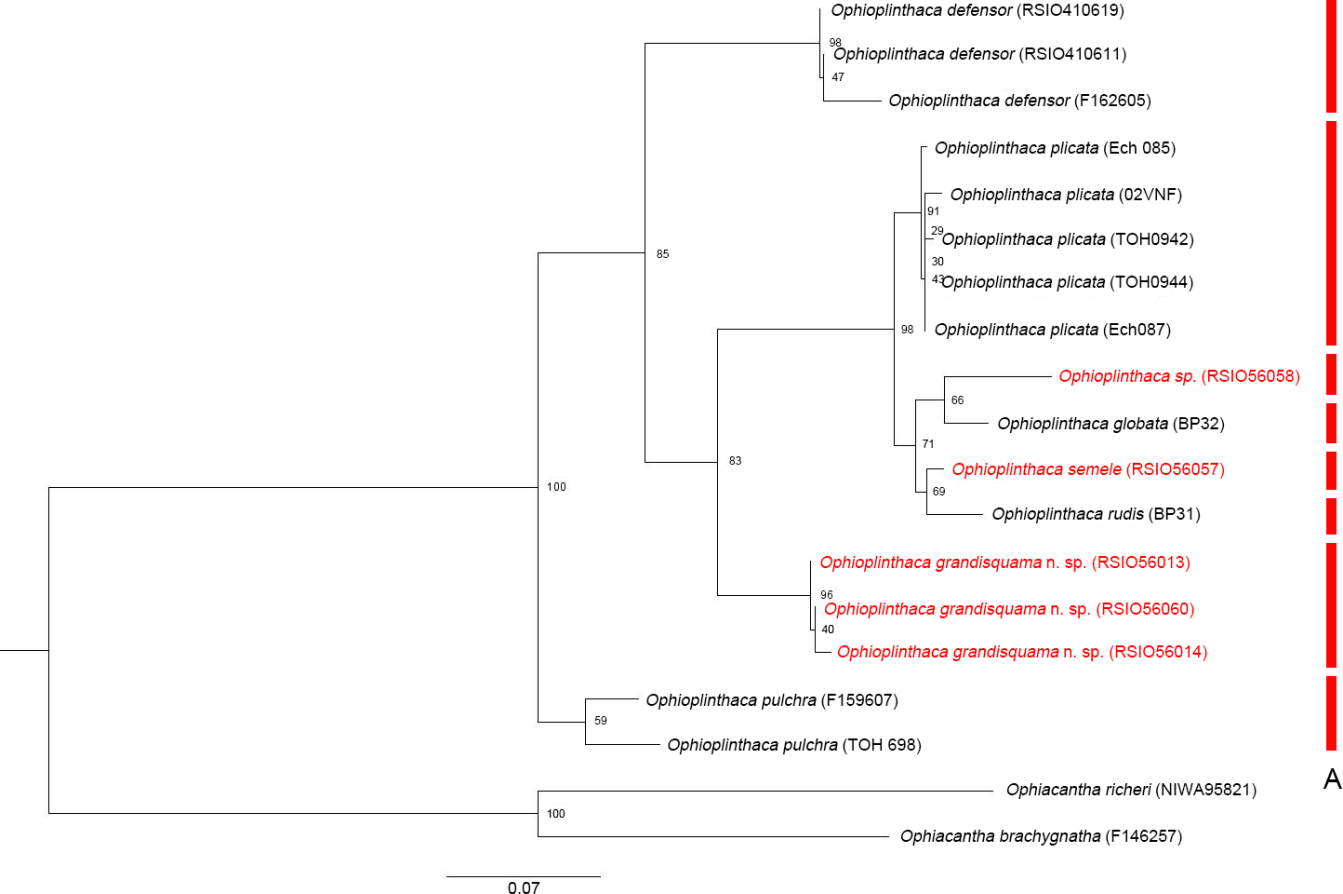
Maximum likelihood tree of the genus *Ophioplinthaca* based on COI sequences. Colored bars in red refer to MOTUs in ABGD.

The phylogenetic analysis (Fig. 13) supported that *O. grandisquama* n. sp. is clearly distinguished from other species of *Ophioplinthaca*, supporting that the three specimens belong to the same species. The maximum likelihood tree showed that *O. semele* was clustered with *O. rudis*, whereas *Ophioplinthaca sp*. was clustered with *O. globata*, together forming a sister clade to *O. plicata*. Although the genetic distances among the four species were low (ranging from 0.032–0.078) (Table 4), the ABGD results supported that they are different species. Additionally, *O. semele* was closely related to *O. rudis*, they can be easily differed from each other based on the morphological characteristics, especially in the shape of disc spines, which are needle-like, long and slender in *O.rudis* instead of cylindrical with obvious thorns on the upper half in *O.semele*. *Ophioplinthaca globata* differs from *O.* sp. in having variable disc spines, many cylindrical to conical, others with only 3 terminal thorns or trifid with bifurcated tips, and radial shields 1/5–1/8 d.d. in length, only contiguous distally or completely separated, whereas in *O.sp*, radial shields 1/4 d.d. in length, contiguous for most of their length, and the disc spines cylindrical to capitate with a terminal crown of thorns.

The intraspecific distances (Table 4) were 0.000–0.012 (*O. plicata*), 0.002–0.009 (*O. grandisquama* n. sp.), 0.001–0.028 (*O. defensor*) and 0.049 (*O. pulchra*). Two previsou studies suggested that the intraspecific distance ranged from 0.005 to 0.064 (Boissin et al., 2017) and from 0.000 to 0.057 (Christodoulou et al., 2020), which were consistent to our results. Three COI sequences of *O. defensor* were used in this analysis, two of which were collected from the northwest Pacific and the other one was collected from the southwest Pacific. The genetic distance was much higher (0.025 and 0.028) between the northwest Pacific and the southwest Pacific than that (0.001) between two specimens from the northwest Pacific. This may be attributed to the large geographical distance, suggesting a potentially distinct population difference of *O. defensor* between the northwest and the southwest Pacific. According to the ABGD species delineation results, the interspecific distances within the genus *Ophioplinthaca* (0.030–0.184, average value 0.117) are also comparable to previous study which suggesting that the average interspecific distances within same genus ranged from 0.056 to 0.316 (Boissin et al., 2017).

**Table 4:** The genetic distance of COI gene (K2P) of Ophioplinthaca.

	1	2	3	4	5	6	7	8	9	10	11	12	13	14	15	16	17	18
1 *Ophioplinthaca pulchra* (MV F159607)																		
2 *Ophioplinthaca pulchra* (MV F159608)	0.049																	
3 *Ophioplinthaca sp.* (RSIO56058)	0.157	0.184																
4 *Ophioplinthaca rudis* (MNHN BP31)	0.160	0.176	0.078															
5 *Ophioplinthaca semele* (RSIO56057)	0.160	0.156	0.060	**0.032**														
6 *Ophioplinthaca plicata* (MV F144759)	0.140	0.148	0.084	0.051	0.036													
7 *Ophioplinthaca plicata* (MV F188868)	0.153	0.160	0.085	0.055	0.042	0.012												
8 *Ophioplinthaca plicata* (MV F144757)	0.134	0.145	0.086	0.050	0.030	0.008	0.009											
9 *Ophioplinthaca plicata* (MV F144758)	0.137	0.155	0.084	0.050	0.036	0.005	0.008	0.004										
10 *Ophioplinthaca globata* (MNHN BP32)	0.146	0.150	**0.063**	0.057	0.050	0.057	0.051	0.032	0.053									
11 *Ophioplinthaca plicata* (MV F144764)	0.125	0.149	0.092	0.054	0.031	0.005	0.003	0.003	0.000	0.000								
12 *O. grandisquama* n. sp.(RSIO56014)	0.123	0.144	0.136	0.125	0.108	0.110	0.115	0.110	0.110	0.107	0.103							
13 *O. grandisquama* n. sp.(RSIO56013)	0.112	0.135	0.141	0.129	0.111	0.114	0.116	0.117	0.114	0.127	0.107	**0.009**						
14 *O. grandisquama* n. sp.(RSIO56060)	0.119	0.148	0.152	0.127	0.108	0.113	0.118	0.120	0.115	0.115	0.117	**0.007**	**0.002**					
15 *Ophioplinthaca defensor* (MV F162605)	0.111	0.147	0.180	0.174	0.138	0.149	0.152	0.153	0.153	0.097	0.152	0.096	0.091	0.119				
16 *Ophioplinthaca defensor* (RSIO410611)	0.122	0.131	0.176	0.159	0.140	0.148	0.153	0.137	0.146	0.151	0.137	0.104	0.103	0.121	0.025			
17 *Ophioplinthaca defensor* (RSIO410619)	0.119	0.129	0.174	0.156	0.137	0.146	0.151	0.135	0.144	0.151	0.134	0.106	0.106	0.123	0.028	0.001		
18 *Ophiacantha richeri* (NIMA95821)	0.270	0.339	0.393	0.357	0.368	0.387	0.350	0.423	0.399	0.300	0.434	0.366	0.349	0.373	0.369	0.350	0.353	
19 *Ophiacantha brachygnatha* (MV F146257)	0.278	0.286	0.346	0.311	0.316	0.316	0.304	0.335	0.312	0.280	0.339	0.307	0.309	0.309	0.324	0.303	0.308	0.202

## Conclusions

Three species of the genus *Ophioplinthaca* were recorded and described, including a new species, *Ophioplinthaca grandisquama* n. sp., which can be easily distinguished from its congeners by the shape and size of tentacle scales and disc spines, as well as radial shields. Morphological characteristics of internal skeleton were also described, providing significant information for future taxonomic study of this genus. Phylogenetic study based on COI supported the delimitation of the new species and the other species with COI sequences available from GenBank in the genus *Ophioplinthaca* in this study. These findings further enrich the distribution of *Ophioplinthaca* from the seamount in the Northwest Pacific Ocean, filling the knowledge gap of benthic invertebrate in the cobalt-rich area.

##  Supplemental Information

10.7717/peerj.11566/supp-1 Supplemental Information 1

10.7717/peerj.11566/supp-2 Supplemental Information 2 COI sequences

10.7717/peerj.11566/supp-3 Supplemental Information 3 MW284978 to MW284982 sequence data

## Additional Information and Declarations

### DNA Deposition

The following information was supplied regarding the deposition of DNA sequences: The COI sequences described here are available at GenBank: MW284978 to MW284982.

### Data Availability

The following information was supplied regarding data availability: DNA sequences of taxa used in phylogenetic analysis are available in the Supplemental File. All specimens (RSIO56013, RSIO56014, RSIO56060, RSIO56057, RSIO56058) were deposited in the Sample Repository of the Second Institute of Oceanography (RSIO), Ministry of Natural Resources, Hangzhou, China.

### New Species Registration

The following information was supplied regarding the registration of a newly described species: Publication LSID: urn:lsid:zoobank.org:pub:A48B7301-0D4B-4280-BF81-639689F923F6. Ophioplinthaca grandisquama n. sp. LSID: urn:lsid:zoobank.org:act:8509E6DB-E902-4A71-9339-EA40725DD688.
