# Supplementary material for: Description of three species of ophioplinthacids, including a new species, from a deep seamount in the Northwest Pacific Ocean
Source: PeerJ. 2021 Jul 2;9:e11566. doi: 10.7717/peerj.11566 (PMC8256812; doi:10.7717/peerj.11566)
Supplement: Supplemental Information 1 [file peerj-09-11566-s001.pdf]

PeerJ Permission Letter

All items (1-5) are required:

1. Title of PeerJ submission: **Description of three species of ophioplinthacids, including a new species, from a deep seamount in the Northwest Pacific Ocean**
2. Printed name of copyright holder: **Lin Shiquan**
3. Select copyrighted item: **Figure**
4. *For figures*: Supply the approved images(s) including the figure number(s) as they appear in the PeerJ submission OR A complete description of the image(s)

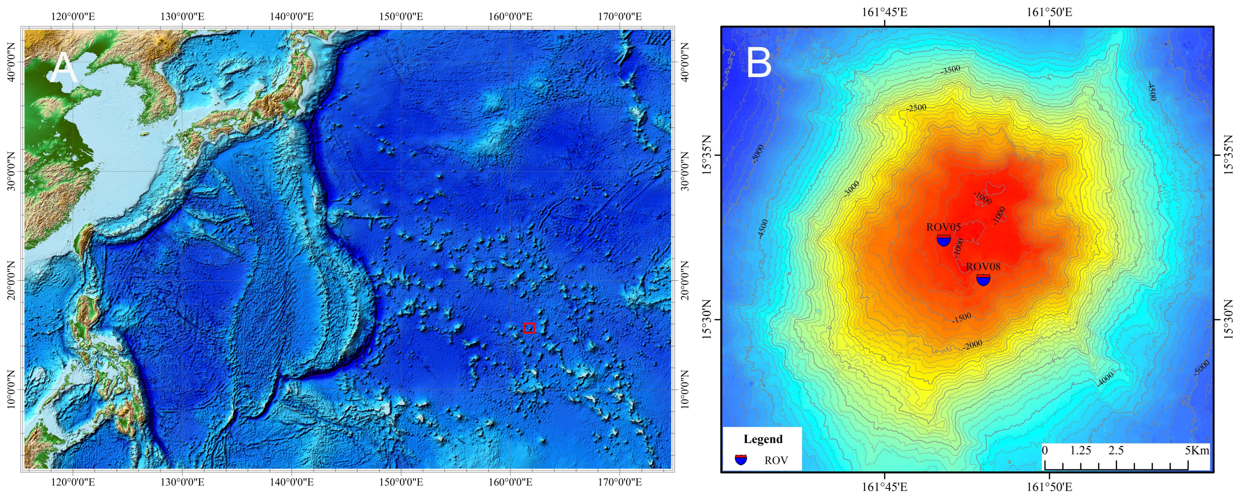

Figure 1 Map of the RC seamount (indicated by the small red block) in the northwest Pacific (A) and sampling sites of specimens of ophioplinthacids (B).

5. I give my permission to PeerJ to publish my work, as described and/or appear below, under the CC-BY 4.0 license.

Lin Shiquan

Signature of copyright holder

May 25, 2021
